# Flexible 2D Cu Metal: Organic Framework@MXene Film Electrode with Excellent Durability for Highly Selective Electrocatalytic NH_3_ Synthesis

**DOI:** 10.34133/2022/9837012

**Published:** 2022-05-30

**Authors:** Jing Wang, Tao Feng, Jiaxin Chen, Jr-Hau He, Xiaosheng Fang

**Affiliations:** ^1^Department of Materials Science, Fudan University, Shanghai 200433, China; ^2^School of Chemical and Environmental Engineering, Shanghai Institute of Technology, Shanghai 201418, China; ^3^Department of Materials Science and Engineering, City University of Hong Kong, Tat Chee Avenue, Kowloon, Hong Kong

## Abstract

Electrocatalytic nitrate reduction to ammonia (ENRA) is an effective strategy to resolve environmental and energy crisis, but there are still great challenges to achieve high activity and stability synergistically for practical application in a fluid environment. The flexible film electrode may solve the abovementioned problem of practical catalytic application owing to the advantages of low cost, light weight, eco-friendliness, simple and scalable fabrication, extensive structural stability, and electrocatalytic reliability. Herein, 2D hybridization copper 1,4-benzenedi-carboxylate (CuBDC) has been grown on electronegative MXene nanosheets (Ti_3_C_2_T_x_) seamlessly to prepare a 2D flexible CuBDC@Ti_3_C_2_T_x_ electrode for ENRA. The flexible electrode simultaneously exhibits high Faradaic efficiency (86.5%) and excellent stability for NH_3_ synthesis, which are comparable to previously reported nanomaterials toward ENRA. Especially, the flexible electrode maintains outstanding *FE*_NH3_ toward ENRA after the bending, twisting, folding, and crumpling tests, indicating excellent electroconductibility, high stability, and durability. This work not only provides mild permeation-mediated strategy to fabricate a flexible electrode but also explores the practical applications of the electrode with effectively environmental adaptability in solving global environmental contamination and energy crisis by effective ENRA.

## 1. Introduction

Ammonia (NH_3_) is a potential hydrogen carrier due to its high energy density (4.32 kW·h/L) and storage capacity (17.6%) [1, 2]. However, it used to rely on the energy-intensive Haber−Bosch process (400–600°C, >400 atm) [3]. Recently, electrocatalytic N_2_ reduction reaction (NRR) has been demonstrated as a potential approach for NH_3_ generation [4] but a large amount of energy could be consumed to break the N≡N bond and the low solubility of N_2_ in water limited the progress of the NRR [5, 6]. The nitrate (NO_3_^−^) is selected as the most viable N source for NH_3_ production due to its high solubility in water and lower energy consumption [7]. In addition, the NO_3_^−^ is one of the most difficult N-pollutants to remove [8–10], which induces global eutrophication and does damage to the human health [11, 12]. Therefore, electrocatalytic NO_3_^−^ reduction to NH_3_ (ENRA) is one of the most promising strategy for producing NH_3_ under ambient conditions and solving global NO_3_^−^ contamination and energy crisis [13, 14]. However, owing to the fragile nature of a traditional electrode, the practical application of ENRA is limited by poor stability and lossy and even disappeared activity after omnidirectional deformability in a fluid environment [14]. Thus, the electrocatalytic NH_3_ synthesis using flexible electrode with effective electrocatalytic activity, high Faradaic efficiency, robust mechanical stability, and low environmental impacts would be a promising strategy to solve the abovementioned problem.

Recently, inspired by the increasing advantages of flexible fuel cell including being lightweight, portable, foldable/twistable, and wearable [15, 16], the two-dimensional (2D) materials have been reported as an ideal choice for constructing a flexible electrode because of the high aspect ratio of the nanostructure, with a transverse size expansion nanometer thickness [17]. Especially, the 2D MXenes have plenty of advantages for electrode preparation, such as high electrical conductivity, flexibility, and hydrophilicity [18, 19]. In addition, MXenes are a kind of materials that can change the electronegativity of the active metal center, thus regulating the performance of the electrode [20, 21]. In addition to the metal-organic frameworks (MOFs), it can serve as a metal center in the catalyst and is an important type of porous coordination compounds [22]. Moreover, 2D MOF nanosheets with the thicknesses of several nanometers are expected to provide the large exposure to active atoms, as well as fast ion mass transfer [17]. For example, 2D copper- (Cu-) based conductive MOF is reported for aqueous CO_2_ reduction reaction at low overpotentials with excellent catalytic activity [23], while NO_3_^−^ reduction can also be catalyzed by the great potential of earth-abundant and cost-effective Cu-based electrode with high activity [24], which is comparable with scarce and high-cost noble metals [25–27]. Thus, the incorporation of 2D Cu MOFs with MXenes could synchronously enhance the ENRA performance, stability, electrical conductivity, flexibility, and eco-friendliness of the electrode [28]. Despite the benefits and demands, there is no report involving flexible electrodes for ENRA.

Based on the abovementioned discussion, in this report, the flexible 2D CuBDC@Ti_3_C_2_T_x_ electrodes are designed by seamlessly coating the 2D CuBDC layer on the Ti_3_C_2_T_x_ film using a simple permeation-mediated strategy under mild conditions (40°C), which are used for ENRA for the first time. The 2D flexible film structure is conducive to produce fully exposed active sites in contact with reaction solution, resulting in outstanding electrocatalytic activity and rapid reaction rate. The flexible electrodes exhibit excellent ENRA performance and mechanical stability. The reaction pathway and mechanism are demonstrated through online differential electrochemical mass spectrometry (DEMS). The following advantages can be achieved through our design: (1) superior mechanical flexibility and outstanding ENRA performance as well as high cyclic stability of the CuBDC@Ti_3_C_2_T_x_, facilitating large-scale practical application of the flexible electrode; (2) comparable ENRA performance to noble metals, promoting their extensive commercialization due to the earth abundance and cost-effectiveness; (3) excellent stability and structural tunability after omnidirectional deformability of the flexible electrode-matching fluid environment for ENRA. For example, when the actual water flow changes greatly, the rigid electrode is easy to cause its efficiency reduction or even damage, while the flexible electrode with structural tunability can adapt to this environment well; and (4) free-standing and lightweight properties for portable electrocatalytic equipment.

## 2. Results and Discussion

**Scheme 1:**
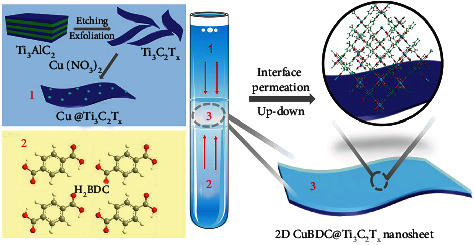
Schematic illustration of the preparation process of the 2D CuBDC@Ti_3_C_2_T_x_ nanosheets as a flexible electrode via permeation-mediated strategy.

The CuBDC@Ti_3_C_2_T_x_ composite system is synthesized via permeation-mediated strategy, and Ti_3_C_2_T_x_ nanosheets can be used as a template for the in situ 2D CuBDC growth. Firstly, the Ti_3_C_2_T_x_ was synthesized from the Ti_3_AlC_2_ precursor in an etching solution containing hydrochloric acid (HCl) and lithium fluoride (LiF) [18]. Further shaking the etched Ti_3_C_2_T_x_ resulted in a colloidal suspension of delaminated Ti_3_C_2_T_x_ nanosheets in the pipe. Then, the 1,4-benzenedicarboxylic acid (H_2_BDC) was placed in the bottom layer of the mixture of N, N-dimethyl formamide (DMF) and acetonitrile (CH_3_CN) solution. Ti_3_C_2_T_x_ nanosheets and Cu (NO_3_)_2_ were put on the upper layer of that solution, and these layers were separated vertically by an intermediate solvent layer according to different densities (Scheme 1). During static permeation, the functional groups (−OH and −F) on Ti_3_C_2_T_x_ nanosheets could absorb Cu^2+^ ions for the formation of Ti_3_C_2_T_x_−Cu via the electrostatic interaction. Then, the Ti_3_C_2_T_x_−Cu and H_2_BDC solutions permeated each other at the intermediate solvent layer where the CuBDC crystals grow slowly on the Ti_3_C_2_T_x_. Finally, CuBDC growth is restricted by the absence of Ti_3_C_2_T_x_−Cu which is in the latent organic phase and CuBDC@Ti_3_C_2_T_x_ nanosheets were removed from the reaction by gravity.

**Figure 1:**
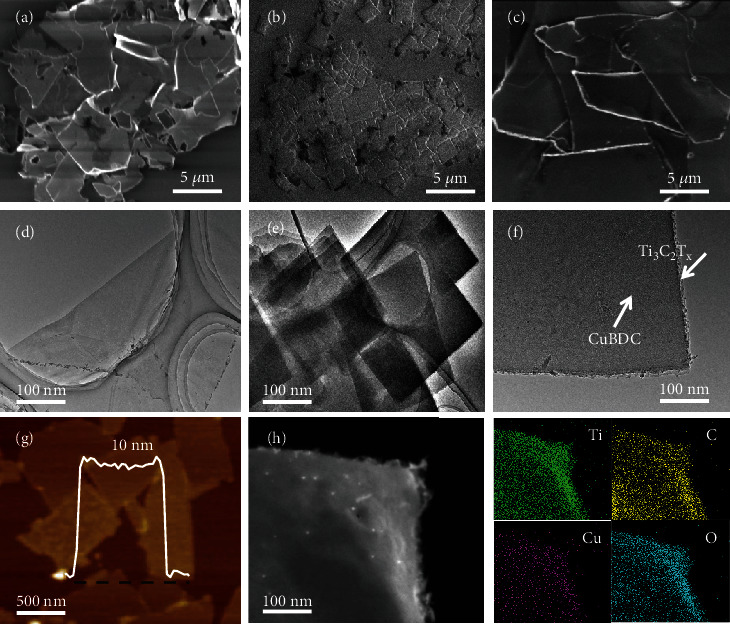
Morphology and structural analysis. SEM images of the (a) Ti_3_C_2_T_x_, (b) CuBDC, and (c) CuBDC@Ti_3_C_2_T_x_ nanosheets. TEM images of the (d) Ti_3_C_2_T_x_, (e) CuBDC, and (f) CuBDC@Ti_3_C_2_T_x_ nanosheets. (g) AFM image, (h) STEM image, and corresponding elemental mapping of Ti, C, Cu, and O of the CuBDC@Ti_3_C_2_T_x_ nanosheets.

**Figure 2:**
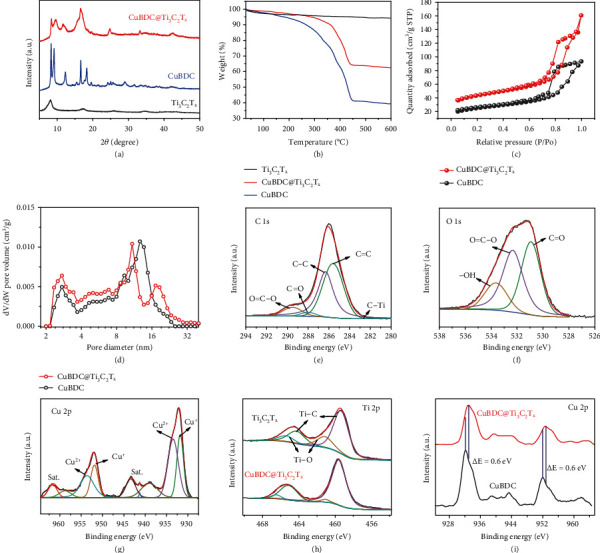
(a) XRD patterns and (b) TGA curves comparison of the Ti_3_C_2_T_x_, CuBDC, and CuBDC@Ti_3_C_2_T_x_ nanosheets. (c) N_2_ adsorption-desorption isotherms and (d) pore size distribution plots for CuBDC and CuBDC@Ti_3_C_2_T_x_ nanosheets. XPS analysis of CuBDC@Ti_3_C_2_T_x_ including (e) C 1s, (f) O 1s, and (g) Cu 2p spectra. The XPS comparison spectra of (h) Ti 2p in the Ti_3_C_2_T_x_ and CuBDC@Ti_3_C_2_T_x_ and (i) Cu 2p in the CuBDC and CuBDC@Ti_3_C_2_T_x_, respectively.

The successful preparation of Ti_3_C_2_T_x_ nanosheets is identified by the scanning electron microscope (SEM) images (Figure 1(a), Figure S1), transmission electron microscopy (TEM) images (Figure 1(d), Figure S2), STEM image, and energy dispersive X-ray spectroscopy (EDS) elemental mapping (Figure S3). The lattice spacing of ~2.6 Å is obtained from the high-resolution transmission electron microscopy (HRTEM), which can be assigned to the (100) plane of Ti_3_C_2_T_x_ (Figure S4). The crystal structures of the exfoliated Ti_3_C_2_T_x_ nanosheets are confirmed by the X-ray diffraction (XRD) pattern (Figure S5). A typical morphological observation by atomic force microscopy (AFM) indicates that the Ti_3_C_2_T_x_ nanosheets have lateral dimensions of several hundreds of nanometers with the thickness of ~3 nm (Figure S6), while the 2D CuBDC nanosheets exhibit a 2D-layered crystalline structure according to the SEM image (Figure 1(b)) and TEM image (Figure 1(e), Figure S7). The diffraction peaks observed in the XRD pattern further verify the crystal structure and composition of CuBDC (Figure 2(a)), which is in accordance with the previous report [17].

Because of the in situ growth of 2D CuBDC, the SEM image of CuBDC@Ti_3_C_2_T_x_ displays the sheet-like morphology with surface roughness (Figure 1(c)). The TEM image of a hybridization nanosheet indicates that CuBDC is coated on the surfaces of Ti_3_C_2_T_x_ nanosheets seamlessly (Figure 1(f)). The AFM image shows square nanosheets with lateral dimensions of 0.6–3 *μ*m and 8–12 nm thicknesses (Figure 1(g)). Furthermore, the STEM image with the corresponding EDS elemental mapping (Figure 1(h)) and the associated atom percentage spectrum (Figure S8 and Table S1) consistently confirm the successful synthesis of the 2D CuBDC@Ti_3_C_2_T_x_ with uniform distribution of Ti, Cu, C, and O elements throughout the nanosheets. In addition, bright particles presented in the STEM image of CuBDC@Ti_3_C_2_T_x_ are Cu nanoparticles. CuBDC is a coordination structure that can be easily destroyed to form Cu nanoparticles under intense electron beam bombardment of STEM.

The XRD patterns of Ti_3_C_2_T_x_, CuBDC, and CuBDC@Ti_3_C_2_T_x_ are depicted in Figure 2(a). The observed diffraction peaks of CuBDC@Ti_3_C_2_T_x_ nanosheets located at 9.4, 11.7, 16.6, 28.8, and 42.4° are assigned to the (001), (222), (333), (442), and (882) planes of CuBDC, respectively [17, 29], while the peak located at 8.2° corresponds to the (002) plane of Ti_3_C_2_T_x_ [18]. Three of the samples are analyzed by thermogravimetric analysis (TGA). As observed from TGA curves (Figure 2(b)), the CuBDC@Ti_3_C_2_T_x_ sample exhibits better thermal stability than that of CuBDC alone, suggesting that the in situ grow strategy significantly enhances the structural stability of the CuBDC@Ti_3_C_2_T_x_. The N_2_ adsorption-desorption isotherms of the CuBDC and CuBDC@Ti_3_C_2_T_x_ reveal type-IV curves with a hysteresis loop (*P*/*P*_0_ = 1), indicating that the pores on the electrocatalysts are dominantly mesopores (Figure 2(c)). As shown in Figure 2(d), the pore size distribution of CuBDC obtained by the density functional theory (DFT) method displays the average pore size range of 3–13 nm. After seamlessly coating with Ti_3_C_2_T_x_, the CuBDC@Ti_3_C_2_T_x_ exhibits a trimodal pore size distribution. Except for the similar mesopores of CuBDC (3 and ~12 nm), the CuBDC@Ti_3_C_2_T_x_ exhibits another mesopore with the size centered at ~20 nm. The results also show that CuBDC@Ti_3_C_2_T_x_ has larger surface areas of 161.45 m^2^/g than that of CuBDC (95.13 m^2^/g). As a result, the CuBDC@Ti_3_C_2_T_x_ shows an enlarged porous structure and specific surface area, which are beneficial to provide plentiful active sites and increase the electrode/electrolyte contact area [30].

The X-ray photoelectron spectroscopy (XPS) survey spectrum of CuBDC@Ti_3_C_2_T_x_ displays the main elements of C, O, Ti, and Cu, and the CuBDC and Ti_3_C_2_T_x_ also are measured as control (Figure S9). For CuBDC@Ti_3_C_2_T_x_, the C 1s spectrum of CuBDC@Ti_3_C_2_T_x_ observes the presence of C−Ti (283 eV), C=C (285.6 eV), C−C (286.5 eV), C=O (288 eV), and O=C−O (289.7 eV) species (Figure 2(e)). Moreover, the O 1s spectrum detects the C=O (531 eV), O=C−O (532.4 eV), and −OH (533.6 eV) species (Figure 2(f)) [20]. Owing to the in situ growth of CuBDC on Ti_3_C_2_T_x_ nanosheets, the change in C and O functional groups can be ignored, which is in line with the fact that Ti_3_C_2_T_x_ kept its structural integrity after CuBDC growth (Figure S10) [31]. Fittings of the Cu 2p peaks of CuBDC@Ti_3_C_2_T_x_ results in the Cu^2+^ component with binding energy peaks located at 934.9 and 954.1 eV while the surface Cu^+^/Cu^0^ species are assigned to 933.4 and 952.7 eV (Figure 2(g)). The Ti 2p XPS spectra of CuBDC@Ti_3_C_2_T_x_ indicate that Ti−C transferred to a higher bonding energy than the pristine Ti_3_C_2_T_x_ (456.2 and 461.8 eV to 459.6 and 465.2 eV) (Figure 2(h)) [21]. This result confirms that the valence state and surface functional groups of Ti in CuBDC@Ti_3_C_2_T_x_ are changed with the gradual growth of CuBDC on the Ti_3_C_2_T_x_ nanosheet, which suggests the strong interaction and charge transfer between Ti_3_C_2_T_x_ and CuBDC [32]. As the oxidation state of Ti can affect the electrical conduction and ENRA performance, Ti−O/Ti−C should be considered properly. To verify the oxidation state of Ti, we have discussed the Ti−O/Ti−C during the CuBDC@Ti_3_C_2_T_x_ synthesis. Ti−O/Ti−C of CuBDC@Ti_3_C_2_T_x_ (21.4%) is lower than that of the origin Ti_3_C_2_T_x_ (8.2%), which is largely because the functional groups (−OH) on Ti_3_C_2_T_x_ nanosheets could absorb Cu^2+^ ions for the formation of Ti_3_C_2_T_x_−Cu via the electrostatic interaction, and then, in situ CuBDC growth occurs. During the generation of CuBDC@Ti_3_C_2_T_x_, part of oxygen-containing groups is occupied, resulting in a decrease in the content of Ti−O. Moreover, the electronic structures of CuBDC and CuBDC@Ti_3_C_2_T_x_ are investigated to identify their intrinsic activity on the electrocatalysis. Compared to pure CuBDC, the Cu 2p_3/2_ XPS spectrum of CuBDC@Ti_3_C_2_T_x_ shifts to lower binding energy by 0.6 eV (Figure 2(i)), which indicates the electron transfer from Ti_3_C_2_T_x_ to Cu at the interface of CuBDC@Ti_3_C_2_T_x_. Therefore, it can be concluded that the higher electron density of Cu leads to a decrease in the barrier reaction and a competitive inhibition of H_2_ production [31], leading to excellent performances of CuBDC@Ti_3_C_2_T_x_ for ENRA.

**Figure 3:**
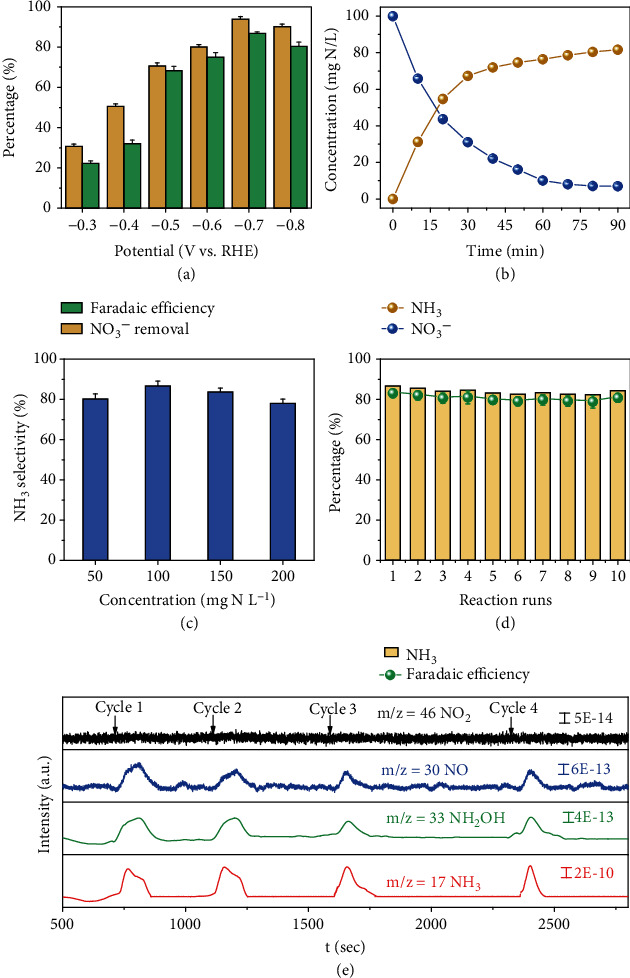
The electrocatalytic NO_3_^−^ reduction to NH_3_ (ENRA) performance based on the CuBDC@Ti_3_C_2_T_x_. (a) The potential-dependent NO_3_^−^ conversion efficiency and Faradaic efficiency, (b) time-dependent concentration change of NO_3_^−^ and NH_3_, (c) NH_3_ selectivity versus different concentrations of NO_3_^−^ at −0.7 V vs RHE, (d) the consecutive recycling tests of ENRA, and (e) DEMS measurements of ENRA of CuBDC@Ti_3_C_2_T_x_ (100 mg·N/L of NO_3_^−^, 0.1 M Na_2_SO_4_).

The electrochemical tests are executed in a double-compartment cell to evaluate the ENRA performance of CuBDC@Ti_3_C_2_T_x_ (Figure S11). Varied potentials from −0.3 to −0.8 vs RHE are applied to choose the optimum one to ensure high performance of ENRA. As shown in Figure 3(a), the maximum NO_3_^−^ conversion efficiency (93.1%) and *FE*_NH3_ (86.5%) are obtained at −0.7 V instead of −0.8 V vs RHE. This result can be attributed to the occurrence of excessive hydrogen evolution reaction (HER) side reaction as indicated by literature results [33]. Thus, −0.7 V has been chosen as the operation voltage of subsequent batch experiment. As shown in Figure 3(b), the concentration of NH_3_ produced on the CuBDC@Ti_3_C_2_T_x_ increases with the rapidly electrocatalytic reduction of NO_3_^−^ below 10 mg·N/L (the maximum contaminant level limited by the World Health Organization [11]) within 50 min. As a comparison, the CuBDC electrode shows a significantly decreased NH_3_ product and Ti_3_C_2_T_x_ exhibits neglectful NH_3_ generation (Figure S12). When NO_3_^−^ concentration increases from 50 to 200 mg·N/L, NH_3_ selectivity remains basically unchanged, which reveals that the concentration is applicable to a wide range of the CuBDC@Ti_3_C_2_T_x_ (Figure 3(c)). The NH_3_ selectivity and Faradaic efficiency retain more than 80% after 10 cycles of ENRA on the CuBDC@Ti_3_C_2_T_x_ (Figure 3(d)). Ti−O/Ti−C of CuBDC@Ti_3_C_2_T_x_ after ENRA reaction (9.3%) (Figure S13) is slightly higher than that of the origin CuBDC@Ti_3_C_2_T_x_ (8.2%). In ENRA reaction, Ti may be oxidized by NO_3_^−^ but the electrons generated in situ in the electrochemical process can further reduce it, thus maintaining the stable state of Ti, which is conducive to maintaining its strong conductivity and is more conducive to the ENRA reaction. These results consistently demonstrated the excellent ENRA performance, high durability, and long-term stability of the CuBDC@Ti_3_C_2_T_x_ electrode.

Direct proof of NO_3_^−^ to NH_3_ during the ENRA is acquired by the online DEMS system. When the applied voltages are varied from 0.1 to −0.9 V vs RHE, signals at m/z values of 46 (NO_2_), 30 (NO), 33 (NH_2_OH), and 17 (NH_3_) appear during four cycles (Figure 3(e)). The presence of m/z signals at 33 shows that typical fragments of NH_2_OH are detected, which is supported by the previous report [24]. m/z of 17 may come from evaporating water, but the water fragments do not change with electric potential. Therefore, it is reasonable to assume that the ENRA process can be traced with the above-obtained signals. Therefore, m/z of 17 is the signal confirming the formation of NH_3_. Based on the abovementioned results, it can be concluded that the reaction pathway of ENRA is as follows: NO_3_^−^→NO_2_^−^→NO→NH_2_OH→NH_3_.

**Figure 4:**
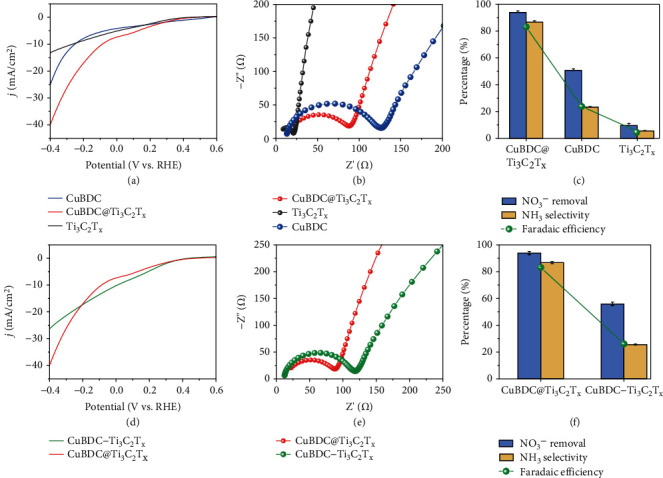
(a) LSV curves, (b) EIS plots, and (c) ENRA performance of the electrodes modified by CuBDC, Ti_3_C_2_T_x_, and CuBDC@Ti_3_C_2_T_x_, respectively. (d) LSV curves, (e) EIS plots, and (f) ENRA performance of the electrodes modified by the physically mixed CuBDC−Ti_3_C_2_T_x_ and CuBDC@Ti_3_C_2_T_x_ (0.1 M Na_2_SO_4_ electrolyte, 100 mg·N/L NO_3_^−^).

With the addition of NO_3_^−^, the linear sweep voltammetry (LSV) curves of CuBDC@Ti_3_C_2_T_x_, CuBDC, and Ti_3_C_2_T_x_ all show the obvious increase in current density (Figure 4(a)). The CuBDC@Ti_3_C_2_T_x_ (−39.8 mA/cm^2^) appears higher current density than those of CuBDC (−24.9 mA/cm^2^) and Ti_3_C_2_T_x_ (−13.1 mA/cm^2^) at −1.0 V vs RHE. The fitting electrochemical impedance spectroscopy (EIS) data elucidate that the CuBDC@Ti_3_C_2_T_x_ generates a smaller arc radius than the CuBDC after adding the Ti_3_C_2_T_x_ nanosheets, pointing to remarkable improved charge transfer kinetics (Figure 4(b)). This result demonstrates that Ti_3_C_2_T_x_ is an ideal 2D nanomaterial to improve the electronic conductivity of the electrode. Compared with those of the Ti_3_C_2_T_x_, the cyclic voltammetry (CV) curves of the CuBDC@Ti_3_C_2_T_x_ and CuBDC show obvious reduction and oxidation peaks (Figure S14). To reveal the advantages of the designed CuBDC@Ti_3_C_2_T_x_, the ENRA performances of the CuBDC and Ti_3_C_2_T_x_ are also conducted for comparison. As shown in Figure 4(c), the CuBDC@Ti_3_C_2_T_x_ obtains higher NO_3_^−^ conversion efficiency, NH_3_ selectivity, and *FE*_NH3_ than CuBDC (51.6%, 22.3%, and 21.8%, respectively) and Ti_3_C_2_T_x_ (9.6%, 5.1%, and 5.3%, respectively). These contrasts consistently demonstrate that the in situ growth of CuBDC on Ti_3_C_2_T_x_ nanosheets not only provides plentiful active sites but also enhances electronic conductivity, leading to accelerated electron transfer and improved electrocatalytic activity for ENRA. In addition, the ENRA performance of CuBDC@Ti_3_C_2_T_x_ is comparable to or even better than other previous reported electrodes (Table S2, S3) [34–39].

Compared with the CuBDC@Ti_3_C_2_T_x_, the LSV curves of CuBDC−Ti_3_C_2_T_x_ exhibit lower current density (Figure 4(d)) and the EIS measurements also show that the larger the arc radius, the worse the charge transfer kinetics (Figure 4(e)). Moreover, the physically mixed CuBDC−Ti_3_C_2_T_x_ shows typical overlap nanosheets (Figure S15) and significantly decreased NO_3_^−^ conversion efficiency (50.7%), NH_3_ selectivity (23.3%), and Faradaic efficiency (22.8%) (Figure 4(f)). The LSV curves of CuBDC@Ti_3_C_2_T_x_ show the obvious increase of the current density in the presence of NO_3_^−^ in 0.1 M Na_2_SO_4_ electrolytes, but no current density is generated in the absence of NO_3_^−^ (Figure S16). Furthermore, the CuBDC@Ti_3_C_2_T_x_ produces higher current density than CuBDC and Ti_3_C_2_T_x_ electrodes with the addition of NO_3_^−^ (Figure S17). These results consistently indicate that the flexible CuBDC@Ti_3_C_2_T_x_ electrode shows specific response to the NO_3_^−^, leading to excellent ENRA performance.

Based on the abovementioned experimental results, the outstanding ENRA performances of the CuBDC@Ti_3_C_2_T_x_ may be due to the synergistic coupling effects of the 2D CuBDC and Ti_3_C_2_T_x_ components. The CuBDC provides plentiful active sites and porous structure while the Ti_3_C_2_T_x_ nanosheets provide a flexible support to prevent the aggregation of catalytic sites, thus increasing the exposure of active surfaces and pore structures. When the CuBDC@Ti_3_C_2_T is used for ENRA, the hydrophilic Ti_3_C_2_T_x_ assists in facilitating the easy access of NO_3_^−^ to the Cu-active species (Figure S18) [32]. Moreover, the Ti_3_C_2_T provides high electronic density, which not only reduces the reaction barrier but also suppresses the competing HER [31]. Thus, the hybridization of CuBDC@Ti_3_C_2_T_x_ can be used for highly selective electrocatalytic synthesis of NH_3_ with high Faradaic efficiency.

**Figure 5:**
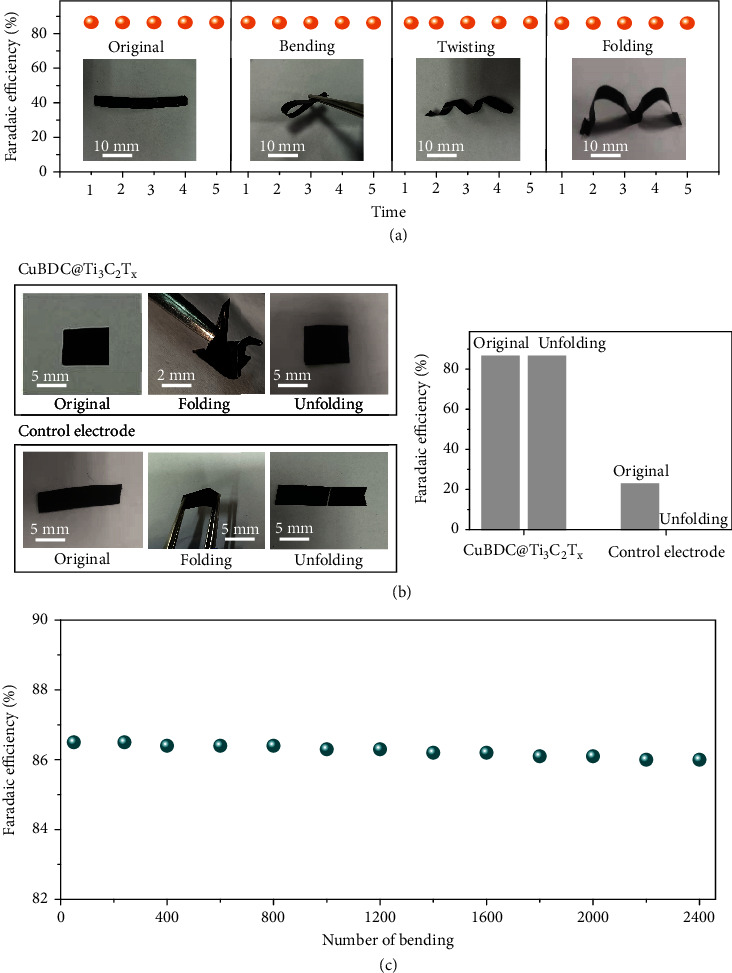
Mechanical deformability of the flexible CuBDC@Ti_3_C_2_T_x_ film electrode. (a) Change in Faradaic efficiency under various deformation modes: original, bending, twisting, and multifolding. (b) Photographs and ENRA performance comparison of flexible CuBDC@Ti_3_C_2_T_x_ electrode versus control electrode under original and unfolding. (c) Vary in Faradaic efficiency under various cycle testing of the flexible electrode after a number of bending.

The mechanical flexibility of the flexible CuBDC@Ti_3_C_2_T_x_ electrode is quantitatively examined under various deformation modes. The flexible CuBDC@Ti_3_C_2_T_x_ electrode retains good ENRA performance after bending, twisting, and folding (Figure 5(a)). This result demonstrates that omnidirectional deformability of the electrode has almost no effect on the ENRA performance. The mechanical flexibility of the CuBDC@Ti_3_C_2_T_x_ electrode is highlighted by comparing it with that of the mixed CuBDC-Ti_3_C_2_T_x_ on the C film (CuBDC-Ti_3_C_2_T_x_-C). Notably, after folding and then unfolding, there is no significant change on the morphology and ENRA performance observed for the flexible CuBDC@Ti_3_C_2_T_x_ electrode, while the CuBDC-Ti_3_C_2_T_x_-C is easily broken and loses electrocatalytic activity (Figure 5(b)). This result indicates that the seamless growth of 2D CuBDC on Ti_3_C_2_T_x_ nanosheets significantly improves mechanical flexibility for extending the electrocatalytic performance of the flexible CuBDC@Ti_3_C_2_T_x_ electrode. Moreover, the CuBDC@Ti_3_C_2_T_x_ film still maintains high *FE*_NH3_ after bending for 2400 cycles, which confirms that the flexible electrode has high stability and durability during application in the fluid environment (Figure 5(c)). As a result, the CuBDC layers provide fully exposed active centers and pore structure for ENRA while the Ti_3_C_2_T_x_ nanosheets endow the electrode with high electrical conductivity and mechanical flexibility, which enable the flexible CuBDC@Ti_3_C_2_T_x_ electrode to own highly electrocatalytic activity and cycling stability.

## 3. Conclusion

In summary, a flexible 2D free-standing CuBDC@Ti_3_C_2_T_x_ electrode with low resistance and tunable morphology is successfully prepared via permeation-mediated strategy. The fabricated film electrode owns the benefits of simple and scalable fabrication, excellent flexibility, extensive structural stability, low cost, light weight, and eco-friendliness for practical electrocatalytic application. The high NO_3_^−^ conversion efficiency, NH_3_ selectivity, and Faradaic efficiency of ENRA are obtained on the flexible electrode, which are comparable to the nanocatalysts based on precious metals previously reported. In particular, the omnidirectional deformability of the film electrode has neglectful effect on the ENRA performance, indicating excellent flexibility, stability, and durability. The reaction mechanism and pathway of the ENRA are demonstrated by the analysis results of online DEMS. This CuBDC@Ti_3_C_2_T_x_ film exhibits a plentiful porous structure, large active surface area, high electrical conductivity, and superb mechanical flexibility, which can significantly improve the electrocatalytic activity and stability for effective environmental adaptability of the flexible electrode toward ENRA in the fluid environment.

## 4. Material and Methods

### 4.1. Materials

Copper nitrate trihydrate (Cu(NO_3_)_2_·3H_2_O, 99%), 1,4-benzenedicarboxylic acid (H_2_BDC, 98%), sodium nitrate (NaNO_3_, 99%), sodium nitrite (NaNO_2_, 99%), ammonium chloride (NH_4_Cl, 99%), sodium sulfate (Na_2_SO_4_, 99%), acetylene black, acetonitrile (CH_3_CN, 99%), NaOH (96%), HCl (36-38%), N, N-dimethylformamide (DMF, 99.5%), sulfamic acid, p-aminobenzenesulfonamide, N-(1-Naphthyl) ethylenediamine dihydrochloride, phosphoric acid, trichloromethane (CHCl_3_, 99%), and Nessler's reagent are from Sinopharm Chemical Reagent Co. Ltd. LiF (98.5%, 325 mesh, Alfa Aesar), Ti_3_AlC_2_ (98%, 400 mesh, 11 Technology Co. Ltd.), and all reagents were of analytical grade, and solutions were prepared by using ultrapure water (specific resistance of 18 M*Ω* cm).

### 4.2. Preparation of Ti_3_C_2_T_x_ Nanosheets

1 g of LiF was added into 20 mL 9 M HCl solution under stirring for 30 min. Subsequently, 1 g of Ti_3_AlC_2_ powder was slowly added into the solution in 5 minutes and the mixed solution was further stirred for 24 h at 40°C. After etching, the resultant solution was separated via centrifugation with washing (10 min at 3500 rpm), and then, the supernatant was decanted, followed by the addition of ultrapure water and 5 min handshaking. The washing process was repeated several times until the supernatant became dark-green and the pH was around 6.

### 4.3. Synthesis of CuBDC

A glass tube with 14 mm of the inner diameter was used to synthesize CuBDC nanosheets. Dissolve 30 mg H_2_BDC in 2 mL DMF and 1 mL CH_3_CN mixture at the bottom of the tube. In this solution, carefully add a mixture of 1 mL DMF and 1 mL CH_3_CN to prevent the premature mixture of the two solutions. Finally, 30 mg Cu(NO_3_)_2_·3H_2_O is dissolved in a mixture of 1 mL DMF and 2 mL CH_3_CN and carefully add it to the top layer tube. After leaving the tube to react in static conditions at 40°C for 24 h, the formation of a blue precipitate was observed at the bottom of the tube, which was collected by centrifugation at 8000 rpm and consecutively washed 3 times with 1 mL of DMF followed by another 3 times with 1 mL of CHCl_3_ and dried in a vacuum oven at 50°C for 12 h.

### 4.4. Fabrication of the Flexible CuBDC@Ti_3_C_2_T_x_ Electrode

The fabrication method for CuBDC@Ti_3_C_2_T_x_ was similar to that of the CuBDC, except that 10 mg of Ti_3_C_2_T_x_ nanosheets along with 30 mg Cu(NO_3_)_2_·3H_2_O was added to the top layer (mixture of 1 mL DMF and 2 mL of CH_3_CN).

### 4.5. Material Characterizations

The SEM (Gemini SEM 500), TEM (Talos F200X), STEM (Talos F200X) with EDS, and AFM measurement (Bruker Dimension Edge atomic force microscope) were conducted to investigate the morphologies of samples. XPS spectra (ESCALAB 250Xi) were measured using the Mg K*α* line as the excitation source. XRD patterns (Bruker D8 ADVANCE) were recorded with an X-ray diffractometer. TGA (Waters Discovery) was introduced to determine the thermal properties in a N_2_ atmosphere in which the heating rate is 5°C/min. N_2_ adsorption–desorption data were obtained from the Micromeritics ASAP2020M analyzer. Density functional theory (DFT) is used to calculate the pore size distribution. The DEMS (QAS 100) data were collected in the online gas analysis.

### 4.6. Electrochemical Measurements

ENRA experiments were conducted on a three-electrode system in H-type membrane-separated electrolyte cells using a CHI 660D electrochemical workstation. Pt wire and Ag/AgCl electrodes were used as counter and reference electrode. All potentials in contrast to reversible hydrogen electrode (RHE) were recorded. Potential *E* was converted to the RHE: *E*(versus RHE)| = *E*(versus Ag/AgCl) + 0.197V + 0.059V × pH. LSV curves were performed at a rate of 100 mV/s from 0 to –1.0 V vs RHE. Current densities were standard in the geometrical area, and the AC impedance technique was employed to study the electrochemical impedance spectra (EIS) with the CuBDC@Ti_3_C_2_T_x_, CuBDC, Ti_3_C_2_T_x_, and CuBDC−Ti_3_C_2_T_x_-based electrodes. The range of frequency was set from 100 MHz to 1000 Hz. Nitrate solutions (20 mL) with varied initial concentrations (50–200 mg·N/L) were prepared using 200 mg·N/L stock solution and then were added into the cathode cell. Na_2_SO_4_ (0.1 M) as the electrolyte was evenly distributed to the cathode and anode cell. Application of the Amperometric i-t technique was conducted at constant potential (ambient conditions within 90 minutes at −0.7 V vs RHE) [40]. All experiments were carried out in triplicate. ENRA experiments of CuBDC, Ti_3_C_2_T_x_, and physical mixed CuBDC−Ti_3_C_2_T_x_ were performed as control to determine the superiority of CuBDC@Ti_3_C_2_T_x_ electrodes under the same conditions.

## Data Availability

All data needed to evaluate the conclusions in the paper are presented in the paper and/or the Supplementary Materials. Additional data related to this paper may be requested from the authors.

## Supplementary Materials

Supplementary Materials Determination of NO_3_^−^-N, determination of NO_2_^−^-N, determination of NH_3_-N, and calculation of the conversion rate, selectivity, and Faradaic efficiency. Figure S1: the SEM image of the Ti_3_C_2_T_x_ nanosheets. Figure S2: the TEM image of the Ti_3_C_2_T_x_ nanosheets. Figure S3: the SEM image and corresponding EDS elemental mapping of Ti, C, O, F, and element overlay of the Ti_3_C_2_T_x_ nanosheets. Figure S4: the HRTEM image of Ti_3_C_2_T_x_ nanosheets with the corresponding SAED pattern. Figure S5: XRD pattern of Ti_3_C_2_T_x_ nanosheets. Figure S6: AFM pattern of the Ti_3_C_2_T_x_ nanosheets. Figure S7: the TEM image of the CuBDC nanosheets. Figure S8: EDS spectrum of the CuBDC@Ti_3_C_2_T_x_ nanosheets. Figure S9: the XPS survey spectra of CuBDC@Ti_3_C_2_T_x_, Ti_3_C_2_T_x_, and CuBDC witness the main elements of Ti, C, O, and Cu. Figure S10: the XPS analysis of Ti_3_C_2_T_x_ including the (a) C 1s, (b) Ti 2p, (c) O 1s, and (d) F 1s spectra. Figure S11: the photo of double-compartment cell for electrocatalytic nitrate reduction to ammonia based on the flexible 2D CuBDC@Ti_3_C_2_T_x_ film electrode. Figure S12: the time-dependent concentration change of NO_3_^−^ and NH_3_ for ENRA reaction based on the (a) CuBDC and (b) Ti_3_C_2_T_x_ electrodes. Figure S13: the XPS comparison spectra of Ti 2p in CuBDC@Ti_3_C_2_T_x_ after ENRA. Figure S14: the CV plots of the CuBDC@Ti_3_C_2_T_x_, CuBDC, and Ti_3_C_2_T_x_ electrodes. Figure S15: the TEM image of the CuBDC−Ti_3_C_2_T_x_ nanosheets. Figure S16: the LSV curves of the electrodes modified by CuBDC@Ti_3_C_2_T_x_ in 0.1 M Na2SO4 electrolytes with or without NO_3_^−^ (100 mg·N/L). Figure S17: LSV curves of (a) CuBDC and (b) Ti_3_C_2_T_x_ in 0.1 M Na_2_SO_4_ electrolytes with or without NO_3_^−^ (100 mg·N/L). Figure S18: schematic illustration of the possible reaction pathways at CuBDC@Ti_3_C_2_T_x_ for ENRA. Table S1: the atom percentage of the CuBDC@Ti_3_C2T_x_ nanosheets. Table S2: comparison of ammonia selectivity by electrocatalytic nitrate reduction reported in the literatures. Table S3: comparison of FENH3 and NH3 yield rates by electrocatalytic nitrate reduction reported in the literatures.
