# Supplementary material for: Flexible 2D Cu Metal: Organic Framework@MXene Film Electrode with Excellent Durability for Highly Selective Electrocatalytic NH3 Synthesis
Source: Research (Wash D C). 2022 May 30;2022:9837012. doi: 10.34133/2022/9837012 (PMC9175116; doi:10.34133/2022/9837012)
Supplement: Supplementary Materials — Determination of NO3−-N, determination of NO2−-N, determination of NH3-N, and calculation of the conversion rate, selectivity, and Faradaic efficiency. Figure S1: the SEM image of the Ti3C2Tx nanosheets. Figure S2: the TEM image of the Ti3C2Tx nanosheets. Figure S3: the SEM image and corresponding EDS elemental mapping of Ti, C, O, F, and element overlay of the Ti3C2Tx nanosheets. Figure S4: the HRTEM image of Ti3C2Tx nanosheets with the corresponding SAED pattern. Figure S5: XRD pattern of Ti3C2Tx nanosheets. Figure S6: AFM pattern of the Ti3C2Tx nanosheets. Figure S7: the TEM image of the CuBDC nanosheets. Figure S8: EDS spectrum of the CuBDC@Ti3C2Tx nanosheets. Figure S9: the XPS survey spectra of CuBDC@Ti3C2Tx, Ti3C2Tx, and CuBDC witness the main elements of Ti, C, O, and Cu. Figure S10: the XPS analysis of Ti3C2Tx including the (a) C 1s, (b) Ti 2p, (c) O 1s, and (d) F 1s spectra. Figure S11: the photo of double-compartment cell for electrocatalytic nitrate reduction to ammonia based on the flexible 2D CuBDC@Ti3C2Tx film electrode. Figure S12: the time-dependent concentration change of NO3− and NH3 for ENRA reaction based on the (a) CuBDC and (b) Ti3C2Tx electrodes. Figure S13: the XPS comparison spectra of Ti 2p in CuBDC@Ti3C2Tx after ENRA. Figure S14: the CV plots of the CuBDC@Ti3C2Tx, CuBDC, and Ti3C2Tx electrodes. Figure S15: the TEM image of the CuBDC−Ti3C2Tx nanosheets. Figure S16: the LSV curves of the electrodes modified by CuBDC@Ti3C2Tx in 0.1 M Na2SO4 electrolytes with or without NO3− (100 mg·N/L). Figure S17: LSV curves of (a) CuBDC and (b) Ti3C2Tx in 0.1 M Na2SO4 electrolytes with or without NO3− (100 mg·N/L). Figure S18: schematic illustration of the possible reaction pathways at CuBDC@Ti3C2Tx for ENRA. Table S1: the atom percentage of the CuBDC@Ti3C2Tx nanosheets. Table S2: comparison of ammonia selectivity by electrocatalytic nitrate reduction reported in the literatures. Table S3: comparison of FENH3 and NH3 yield rates by electrocatalytic nitrat [file 9837012.f1.docx]

Flexible 2D Cu Metal–organic Framework@MXene Film Electrode with Excellent Durability for Highly Selective Electrocatalytic NH_3_ Synthesis

Jing Wang,^1^ Tao Feng,^2^ Jiaxin Chen,^1^ Jr-Hau He,^3*^ Xiaosheng Fang^1*^

^1^ Department of Materials Science, Fudan University, Shanghai, 200433, P. R. China E-mail: [xshfang@fudan.edu.cn](mailto:xshfang@fudan.edu.cn)

^2^ School of Chemical and Environmental Engineering, Shanghai Institute of Technology, Shanghai, 201418, P. R. China

^3^ Department of Materials Science and Engineering, City University of Hong Kong, Tat Chee Avenue Kowloon, Hong Kong. E-mail: [jrhauhe@cityu.edu.hk](mailto:jrhauhe@cityu.edu.hk)

**Determination of NO_3_^−^-N**

1 mL 1M HCl and 0.1 mL 0.8 wt% sulfamic acid solution were added into the diluted electrolyte (50 mL). The absorbance value was calculated by A=A220 nm-2A275 nm (UV-vis spectrophotometer). The concentration-absorbance curve was calibrated with standard NaNO_3_ solutions.

**Determination of NO_2_^−^-N**

1 mL color reagent (A mixture of 4g p-aminobenzenesulfonamide, 0.2 g N-(1-Naphthyl) ethylenediamine dihydrochloride, 10 mL H_3_PO_4_, and 50 mL ultrapure water) was added into the diluted electrolyte (25 mL), and then measured at 540 nm. The concentration-absorbance curve was calibrated with standard NaNO_2_ solutions.

**Determination of** **NH_3_-N**

1 mL potassium sodium tartrate solution and 0.1 mL Nessler’s reagent were added into the diluted electrolyte (50 mL), and then measured at 420 nm. The concentration-absorbance curve was calibrated with standard NH_4_Cl solutions.

**Calculation of the conversion rate, selectivity, and Faradaic efficiency**

For nitrate electroreduction, the conversion rate can be calculated as follows:

$$R_{N{O_{3}}^{-}}=\frac{(N{O_{3}}^{-})_{0}-(N{O_{3}}^{-})_{f}}{(N{O_{3}}^{-})_{0}}\times\text{100\% }$$

The selectivity of NH_3_ was obtained by:

$$S_{{NH}_{3}}=\frac{(N{H_{4}}^{+})_{f}}{(N{O_{3}}^{-})_{0}-(N{O_{3}}^{-})_{f}}\times\text{100\% }$$

The *FE* was defined by electric charge for NH_3_ synthesis and total charge transfer through the surface of the electrode:

$$FE\left( \% \right)=\frac{Q_{exp}}{Q_{theor}}\times\text{100\%=}\frac{n\times F\times N_{i}}{60\times I\times t}\times\text{100\%}$$

the NH_3_ yield rate was calculated by:

$${Yield}_{NH_{3}}=\frac{C_{NH_{3}}\times V}{M_{NH_{3}}\times t\times S}\times\text{100\% }$$

Where $(N{O_{3}}^{-})_{0}$denote the initial concentrations of NO_3_^−^, $(N{O_{3}}^{-})_{f}$and $(N{H_{4}}^{+})_{f}$represent the final concentrations of NO_3_^−^ and NH_4_^+^. *Q_exp_* is experimental charge consumed in the target reaction; *Q_theor_* is the total charge of the reaction. *n* is the number of electrons/mole of the product; *F* is Faraday constant (96487 C/mol); *N_i_* is the amount of the product for ENRA. *I* is applied current (A); *t* is time (min); 60 is the unit conversion factor (60 s/min).


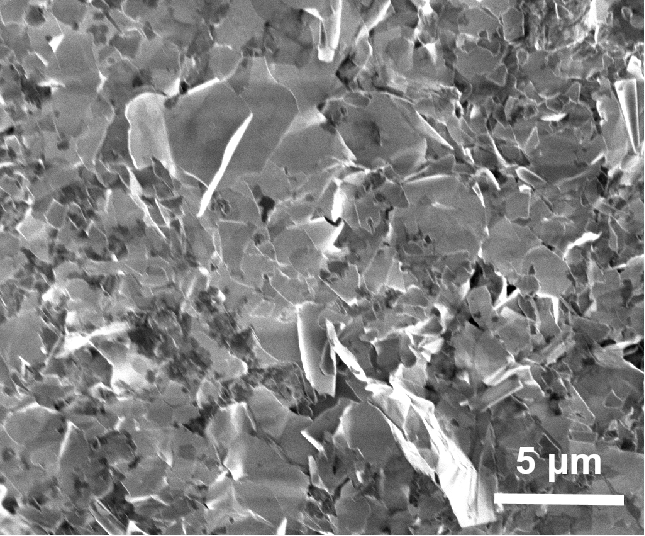


**Figure S1** The SEM image of the Ti_3_C_2_T_x_ nanosheets.

**
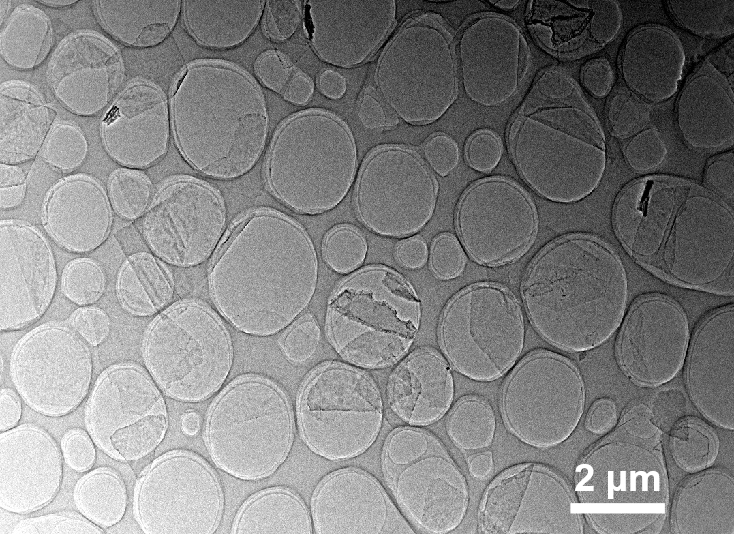
**

**Figure S2** The Transmission Electron Microscopy image of the Ti_3_C_2_T_x_ nanosheets.


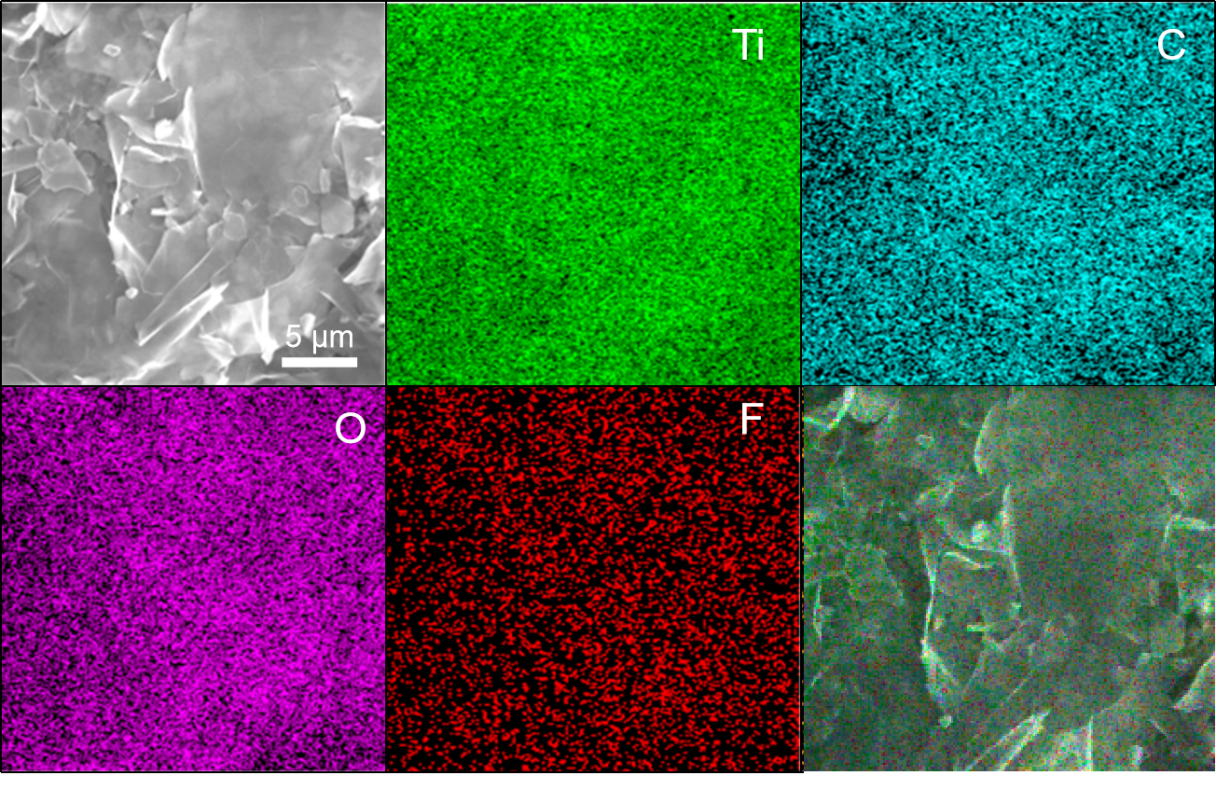


**Figure S3** The SEM image and EDS elemental mapping of Ti, C, O, F and elements overlay of the Ti_3_C_2_T_x_ nanosheets.


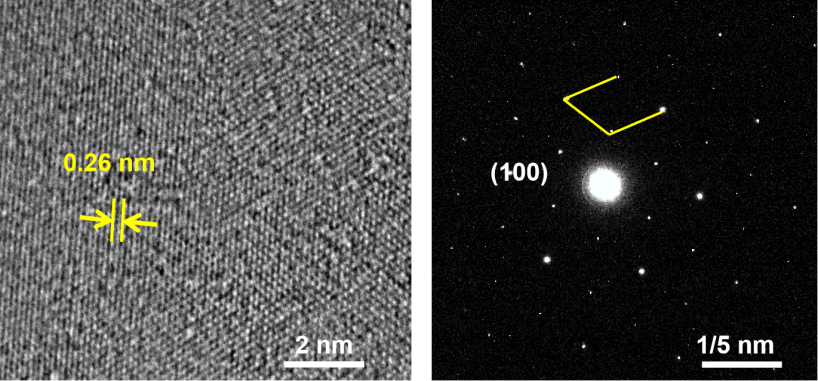


**Figure S4** The HRTEM image of Ti_3_C_2_T_x_ nanosheets with corresponding SAED pattern.


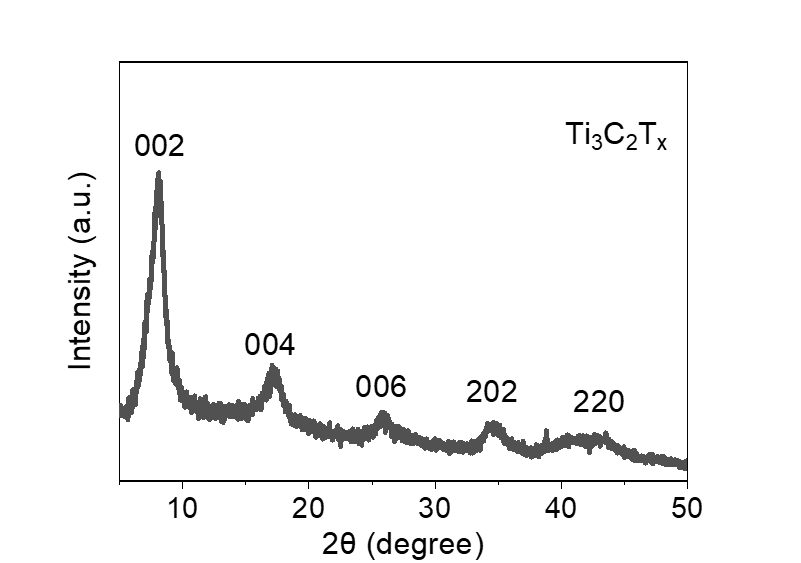


**Figure S5** XRD pattern of Ti_3_C_2_T_x_ nanosheets.


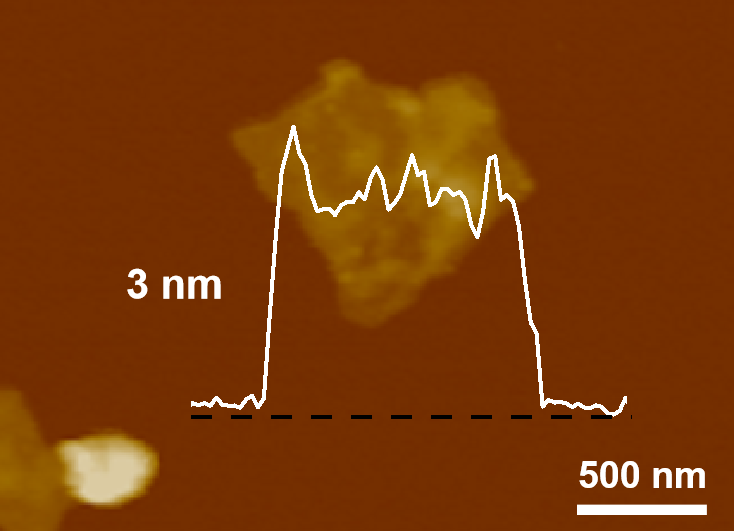


**Figure S6** AFM pattern of the Ti_3_C_2_T_x_ nanosheets.


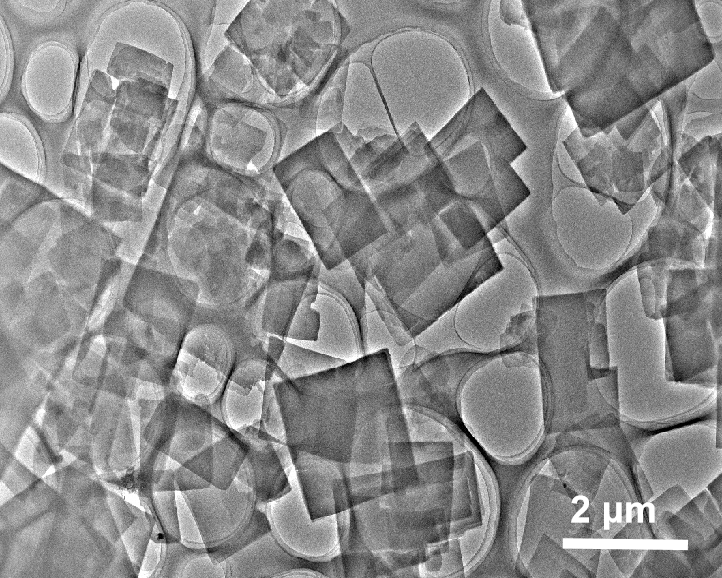


**Figure S7** The TEM image of the 2D CuBDC nanosheets.

**Figure S8** The EDS spectrum of the CuBDC@Ti_3_C_2_T_x_ nanosheets.


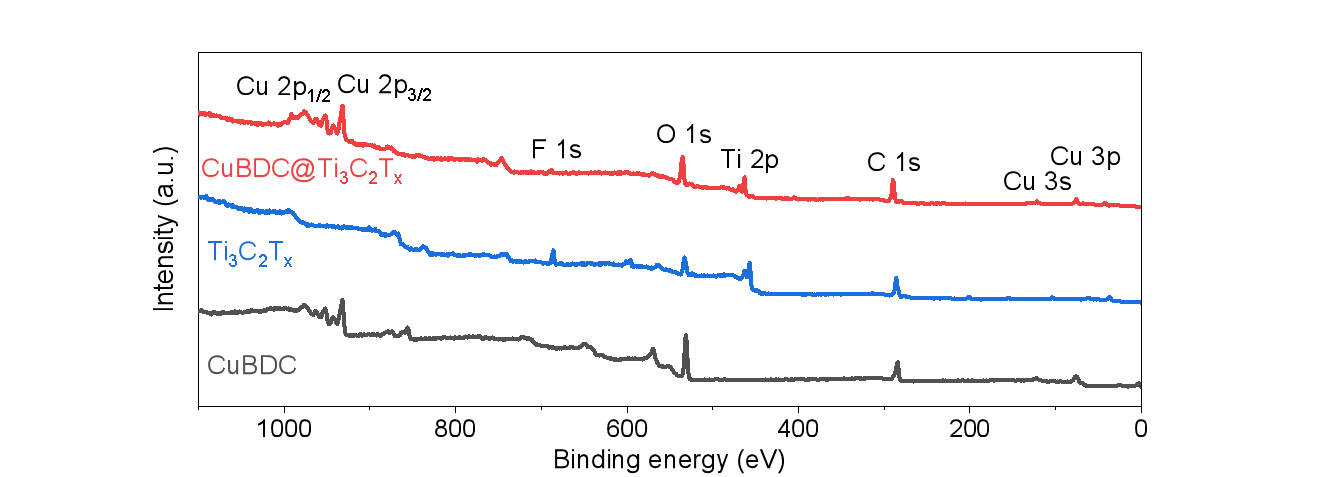


**Figure S9** The XPS survey spectra of CuBDC@Ti_3_C_2_T_x_, Ti_3_C_2_T_x_ and CuBDC witness the main elements of Ti, C, O, and Cu.


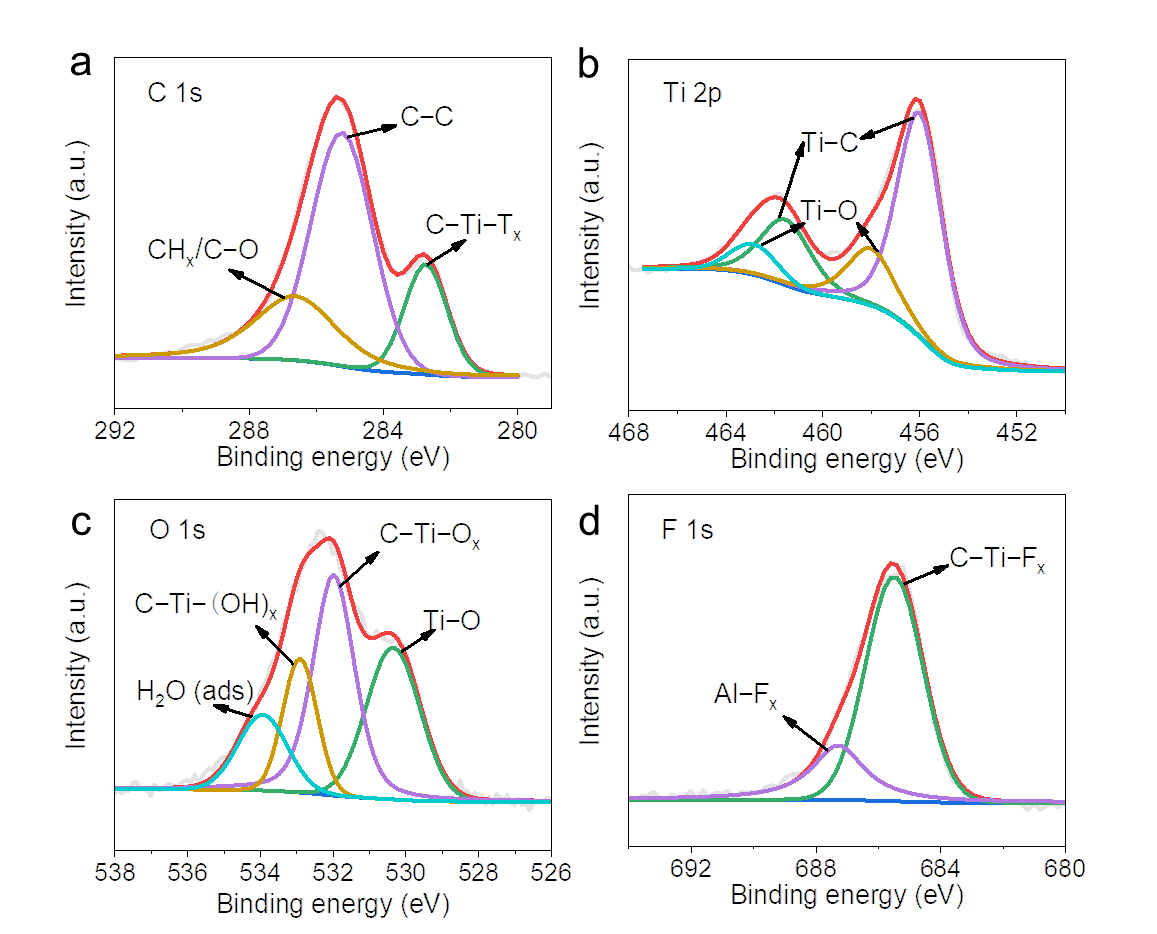


**Figure S10** The XPS analysis of Ti_3_C_2_T_x_ including (a) C 1s, (b) Ti 2p, (c) O 1s and (d) F 1s spectra.


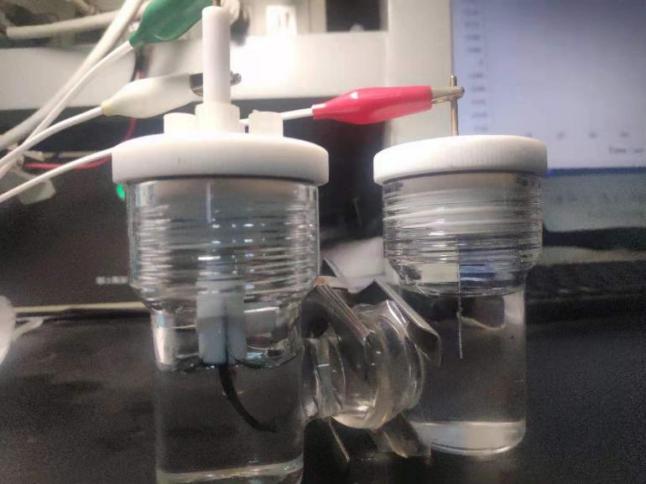


**Figure S11** The photo of double-compartment cell for electrocatalytic nitrate reduction to ammonia based on the flexible 2D CuBDC@Ti_3_C_2_T_x_ film electrode.


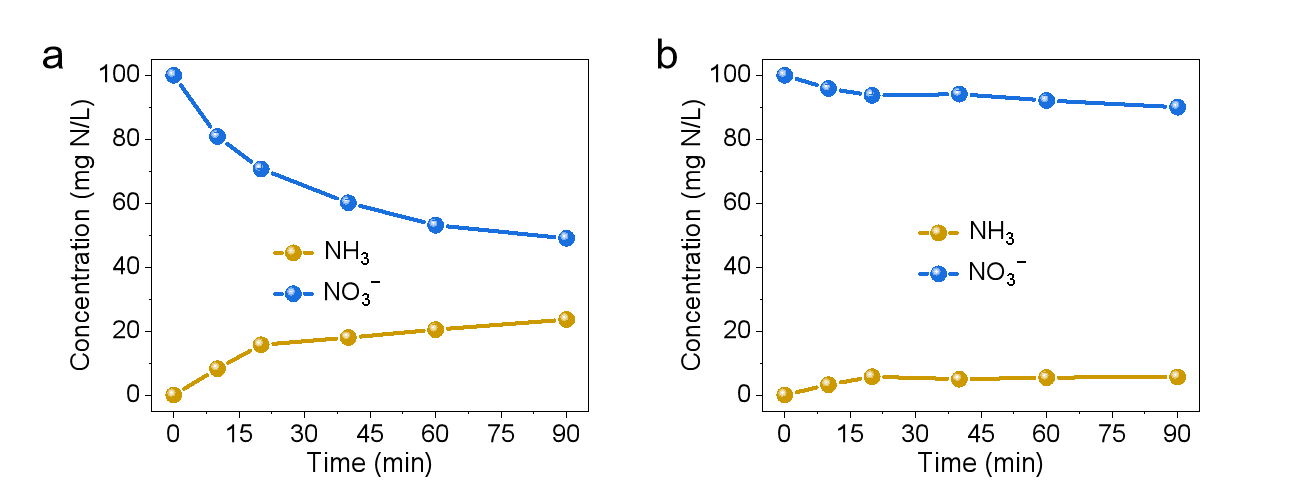


**Figure S12** The time-dependent concentration changes of NO_3_^−^ and NH_3_ for ENRA reaction based on the (a) CuBDC and (b) Ti_3_C_2_T_x_ electrodes.


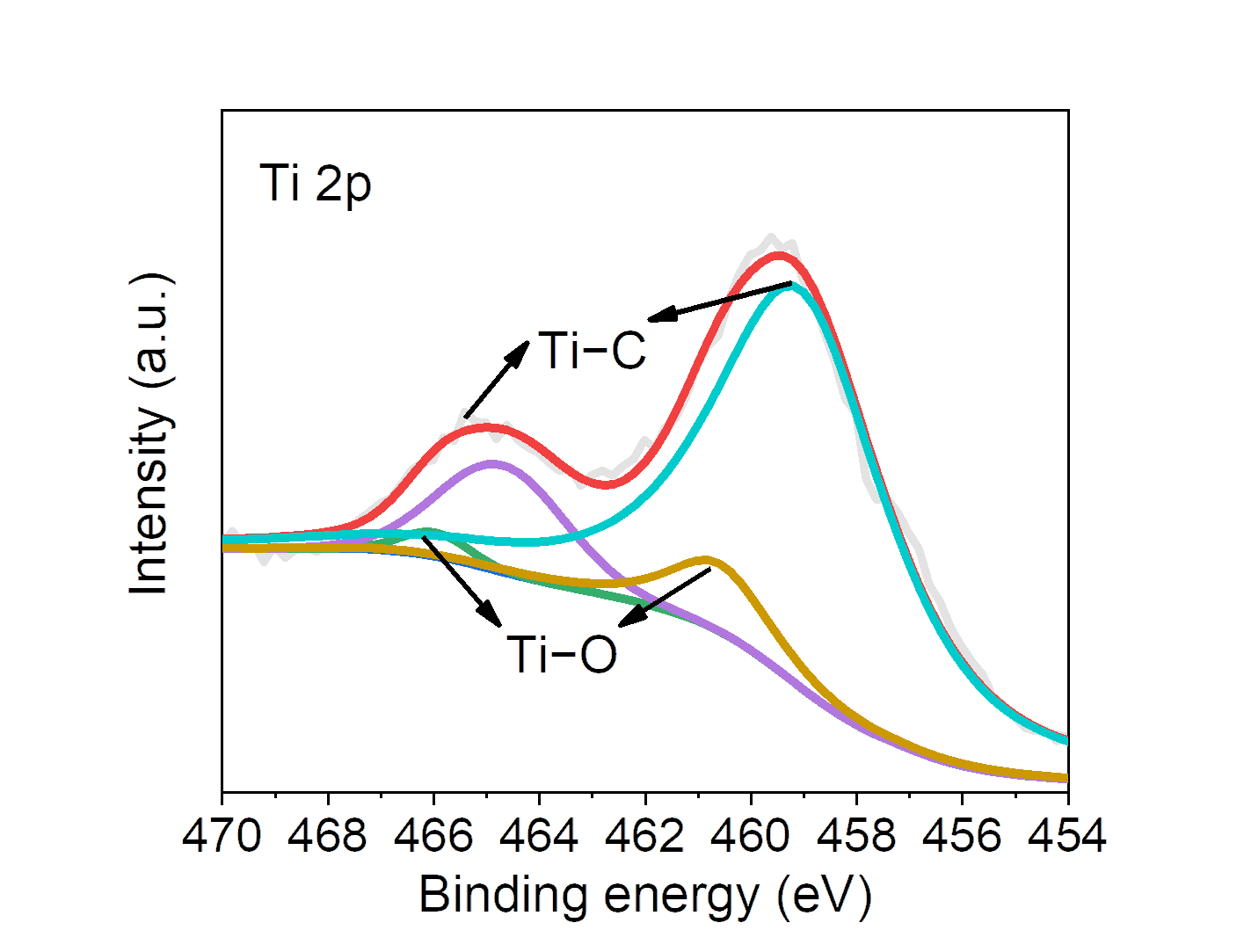


**Figure S13** The XPS comparison spectra of Ti 2p in CuBDC@Ti_3_C_2_T_x_ after ENRA.


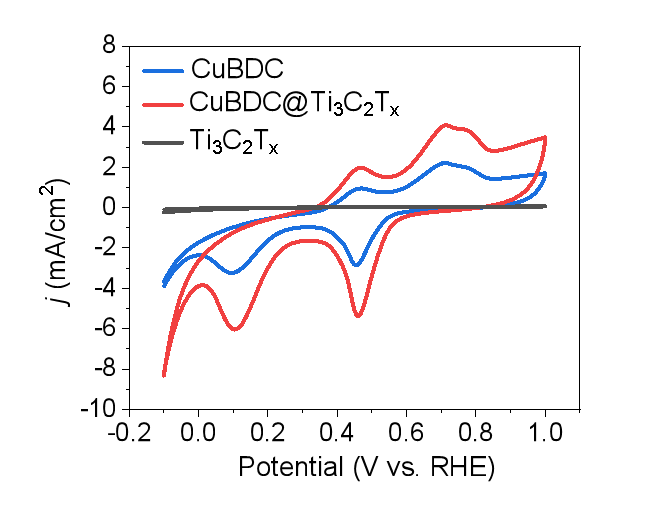


**Figure S14** the CV curves of the CuBDC@Ti_3_C_2_T_x_, CuBDC and Ti_3_C_2_T_x_.


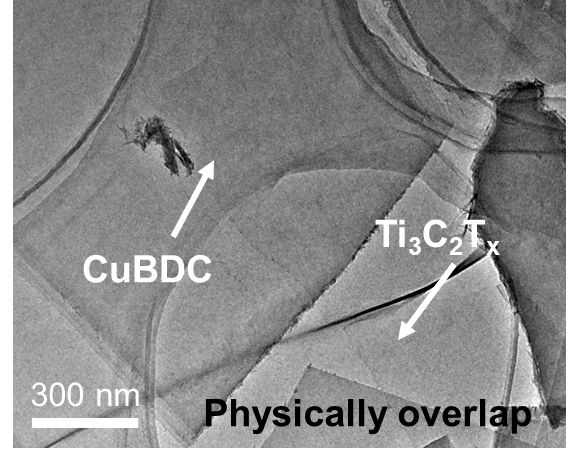


**Figure S15** The TEM image of the CuBDC−Ti_3_C_2_T_x_ nanosheets.


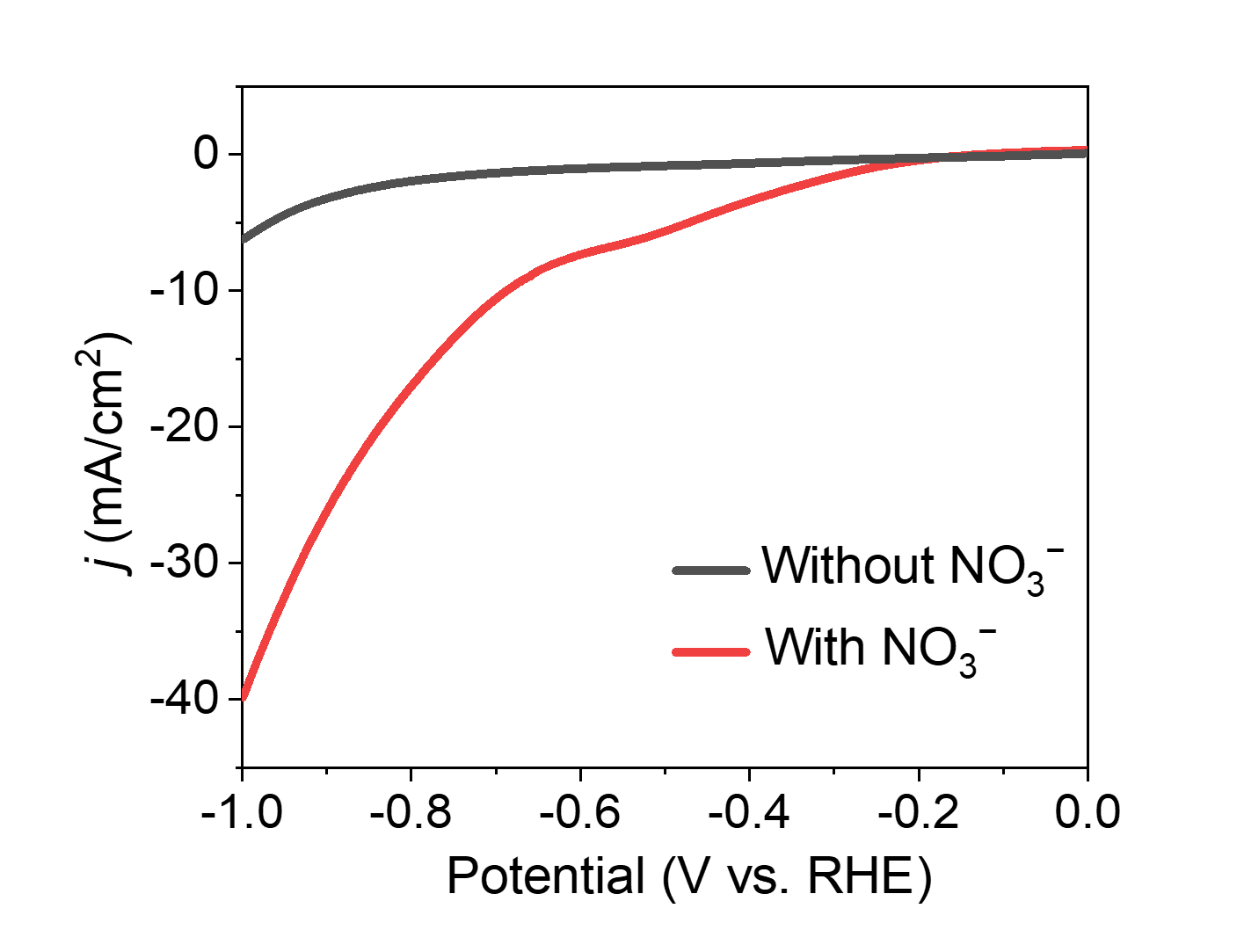


**Figure S16** the LSV curves of the electrodes modified by CuBDC@Ti_3_C_2_T_x_ in 0.1 M Na_2_SO_4_ electrolytes with NO_3_^−^ (red) or without NO_3_^−^ (black).


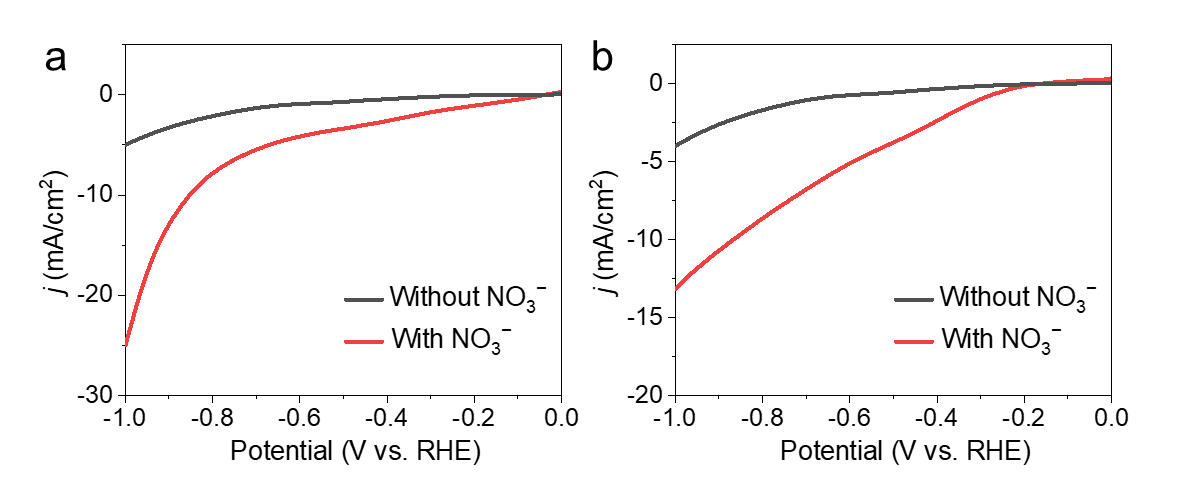


**Figure S17** LSV curves of (a) CuBDC and (b) Ti_3_C_2_T_x_ in 0.1 M Na_2_SO_4_ electrolytes with (red) or without NO_3_^−^ (black).

**
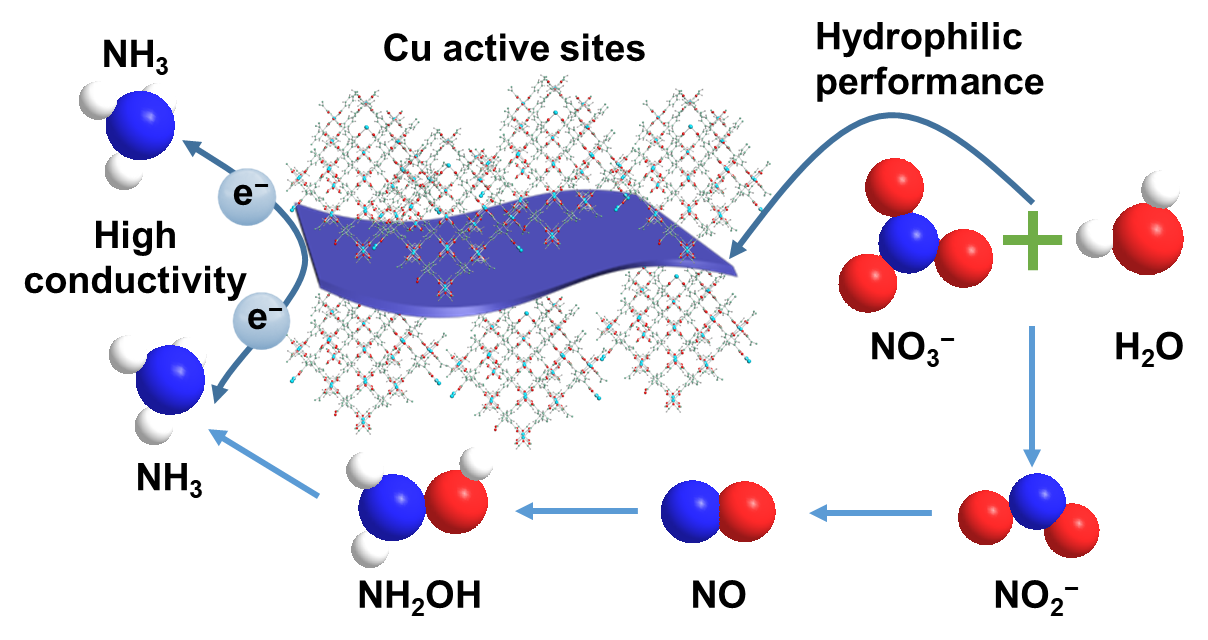
**

**Figure S18** Schematic illustration of possible reaction pathways at CuBDC@Ti_3_C_2_T_x_ for ENRA.

Table S1 The atom percentage of the CuBDC@Ti_3_C_2_T_x_ nanosheets.

| Element | Line Type | Atomic % |
| --- | --- | --- |
| C | K series | 51.88 |
| O | K series | 31.07 |
| Ti | K series | 10.77 |
| Cu | K series | 6.29 |

**Table S2** Comparison of ammonia selectivity by electrocatalytic nitrate reduction reported in the literatures.

| Electrocatalysts | Concentration (mg N L^−1^) | NO_3_^−^ removal  (%) | NH_3_ selectivity  (%) | References |
| --- | --- | --- | --- | --- |
| TiO_2_ | 50 | 35.7 | 85 | [1] |
| Co_3_O_4_ | 50 | 84 | 80 | [2] |
| Cu_80_Ni_20_ | 20 mM | 50 | 88 | [3] |
| Cu/rGO/GP | 280 | 69.8 | 29.9 | [4] |
| CuO NWAs | 200 | 97 | 81.2 | [5] |
| Co_3_O_4_-TiO_2_/Ti | 50 | 89 | 24 | [6] |
| **CuBDC@Ti_3_C_2_T_x_** | **100** | **93.1** | **87.6** | **This work** |

Table S3 Comparison of *FE*_NH3_ and NH_3_ yield rate by electrocatalytic nitrate reduction reported in the literatures.

| Electrocatalysts | *FE*_NH3_ (%) | NH_3_ yield rate | References |
| --- | --- | --- | --- |
| TiO_2-x_ | 85.0% | 0.045 mmol h^-1^ mg^-1^ | [7] |
| Fe_3_Mo_3_C/C | 14.74% | 13.55 μg h^-1^ cm^-2^ | [8] |
| CoS_2_/NS-G | 25.9% | 25.0 μg h^-1^ mg^-1^ | [9] |
| BiNCs | 66% | 52 μmol h^-1^ cm^-2^ | [10] |
| PC/Sb/SbPO_4_ | 31% | 25 μg h^-1^ mg^-1^ | [11] |
| Fe_2_O_3_/CNT | 0.15% | 0.013 μmol h^-1^ cm^-2^ | [12] |
| **CuBDC@Ti_3_C_2_T_x_** | **86.5%** | **0.064 mmol h^-1^ cm^-2^** | **This work** |

**References**

[1] L. Wang, M. Li, C. Feng, W. Hu, G. Ding, N. Chen, X. Liu, "Ti nano electrode fabrication for electrochemical denitrification using Box–Behnken design," *Journal of Electroanalytical Chemistry,* vol. 773, pp. 13-21, 2016.

[2] C. Li, K. Li, C. Chen, Q. Tang, T. Sun, J. Jia, "Electrochemical removal of nitrate using a nanosheet structured Co_3_O_4_/Ti cathode: Effects of temperature, current and pH adjusting," *Separation and Purification Technology,* vol. 237, pp. 116485, 2020.

[3] L. Mattarozzi, S. Cattarin, N. Comisso, P. Guerriero, M. Musiani, L. Vázquez-Gómez, E. Verlato, "Electrochemical reduction of nitrate and nitrite in alkaline media at CuNi alloy electrodes," *Electrochimica Acta,* vol. 89, pp. 488-496, 2013.

[4] D. Yin, Y. Liu, P. Song, P. Chen, X. Liu, L. Cai, L. Zhang, "In situ growth of copper/reduced graphene oxide on graphite surfaces for the electrocatalytic reduction of nitrate," *Electrochimica Acta,* vol. 324, pp. 134846, 2019.

[5] Y. Wang, W. Zhou, R. Jia, Y. Yu, B. Zhang, "Unveiling the activity origin of a copper-based electrocatalyst for selective nitrate reduction to ammonia," *Angewandte Chemie International Edition,* vol. 59, pp. 1-6, 2020.

[6] W. Li, C. Xiao, Y. Zhao, Q. Zhao, R. Fan, J. Xue, "Electrochemical reduction of high-concentrated nitrate using Ti/TiO_2_ nanotube array anode and Fe cathode in dual-chamber cell," *Catalysis Letters,* vol. 146, pp. 2585-2595, 2016.

[7] R. Jia, Y. Wang, C. Wang, Y. Ling, Y. Yu, B. Zhang, "Boosting selective nitrate electroreduction to ammonium by constructing oxygen vacancies in TiO_2_," *ACS Catalysis,* vol. 10, pp. 3533-3540, 2020.

[8] H. Cheng, P. Cui, F. Wang, L.X. Ding, H. Wang, "High Efficiency Electrochemical Nitrogen Fixation Achieved with a Lower Pressure Reaction System by Changing the Chemical Equilibrium," *Angewandte Chemie International Edition,* vol. 58, pp. 15541-15547, 2019.

[9] P. Chen, N. Zhang, S. Wang, T. Zhou, Y. Tong, C. Ao, W. Yan, L. Zhang, W. Chu, C. Wu, Y. Xie, "Interfacial engineering of cobalt sulfide/graphene hybrids for highly efficient ammonia electrosynthesis," *Proceedings of the National Academy of Sciences of the United States of America,* vol. 116, pp. 6635-6640, 2019.

[10] D. Macfarlane, A. Simonov, B.H.R. Suryanto, M. Chatti, H.-L. Du, J. Choi, "Promoting Nitrogen Electroreduction to Ammonia with Bismuth Nanocrystals and Potassium Cations in Water," *Nature Catalysis,* vol. 2, pp. 448-456, 2019.

[11] X. Liu, H. Jang, P. Li, J. Wang, Q. Qin, M.G. Kim, G. Li, J. Cho, "Antimony-Based Composites Loaded on Phosphorus-Doped Carbon for Boosting Faradaic Efficiency of the Electrochemical Nitrogen Reduction Reaction," *Angewandte Chemie International Edition,* vol. 58, pp. 13329-13334, 2019.

[12] S. Chen, S. Perathoner, C. Ampelli, C. Mebrahtu, D. Su, G. Centi, "Electrocatalytic synthesis of ammonia at room temperature and atmospheric pressure from water and nitrogen on a carbon-nanotube-based electrocatalyst," *Angewandte Chemie International Edition,* vol. 56, pp. 2699-2703, 2017.
